# Supplementary figures and images for: Spontaneous reoccurrence of “scooping”, a wild tool-use behaviour, in naïve chimpanzees
Source: PeerJ. 2017 Sep 22;5:e3814. doi: 10.7717/peerj.3814 (PMC5611899; doi:10.7717/peerj.3814)

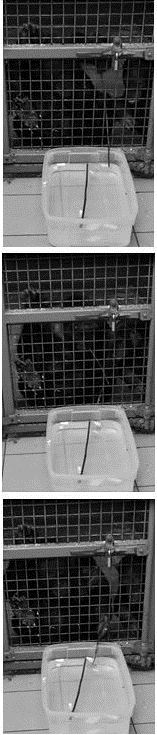

Supplement: Figure S1 — Stick modification process by HO (camera stills by EB). [file peerj-05-3814-s001.tif]

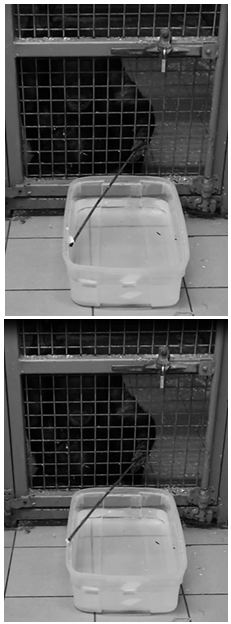

Supplement: Figure S2 — LO using the side technique (camera stills by EB). [file peerj-05-3814-s002.tif]
