# Supplementary material for: Spontaneous reoccurrence of “scooping”, a wild tool-use behaviour, in naïve chimpanzees
Source: PeerJ. 2017 Sep 22;5:e3814. doi: 10.7717/peerj.3814 (PMC5611899; doi:10.7717/peerj.3814)
Supplement: Table S1 [file peerj-05-3814-s003.docx]

| **Name** | **Sex** | **DoB** | **Birth Type** | **Rearing** |
| --- | --- | --- | --- | --- |
| Flyn | M | 25.10.1986 | Captive born | Hand |
| Jomar | M | 28.12.1990 | Captive born | Hand |
| Lottie | F | 28.09.1978 | Captive born | Hand |
| Josie | F | 20.02.1988 | Captive born | Hand |
| Victoria | F | 18.08.1990 | Captive born | Hand |
| Genet | F | 25.05.1995 | Captive born | Hand |
| Tuli | F | 10.08.2007 | Captive born | Parent |
| Tojo | F | From:17.06-12.1997 | Wild | Unknown |
| Coco | F | From:1.1.-31.12.1965 | Wild | Unknown |

S1 Table. **Data on Subjects in Group 1**
