# Supplementary material for: Spontaneous reoccurrence of “scooping”, a wild tool-use behaviour, in naïve chimpanzees
Source: PeerJ. 2017 Sep 22;5:e3814. doi: 10.7717/peerj.3814 (PMC5611899; doi:10.7717/peerj.3814)
Supplement: Table S2 [file peerj-05-3814-s004.docx]

| **Name** | **Sex** | **DoB** | **Birth Type** | **Rearing** |
| --- | --- | --- | --- | --- |
|  |  |  |  |  |
| William | M | 30.06.1982 | Captive born | Unknown |
| Peter | M | 9.07.1992 | Captive born | Parent |
| Samantha | F | From:1.1-31.12.1980 | Wild | Unknown |
| Holly | F | 27.12.1982 | Captive born | Parent |
| Jollie | F | 16.09.1983 | Captive born | Hand |

S2 Table. **Data on Subjects in Group 2**
