# Supplementary material for: Spontaneous reoccurrence of “scooping”, a wild tool-use behaviour, in naïve chimpanzees
Source: PeerJ. 2017 Sep 22;5:e3814. doi: 10.7717/peerj.3814 (PMC5611899; doi:10.7717/peerj.3814)
Supplement: Table S3 [file peerj-05-3814-s005.docx]

S3 Table. Selection of relevant questionnaire responses from the keepers detailing the subjects’ previous tool-use experience (non tool-use behaviours are not included here). Table contains a summary of all the responses provided by the keepers, and behaviours were organised into the relevant action categories.

| **Action/Behaviour** | **Chimpanzee experience as reported by keepers (2015)** |
| --- | --- |
|  | |
| Smashing behaviour | Individuals seen to manipulate stones, but never used them to smash or crack open objects. Stones are immediately removed from the enclosure. |
| Sponging | Individuals have been observed to insert pieces of straw into water dripping from their water dispensers. |
| Stick Dipping | Individuals have been observed to insert sticks into crevices around the enclosure (e.g. in the ground, in the walls). |
| Stick ‘Scooping’ (here we asked specifically for ‘swivelling’ action of the wrist as described by Humle et al., 2011) | No observations of using sticks, or other tools to retrieve food or objects from bodies of water (e.g. puddles). |
| Opening with a Stick | Some individuals have been observed to use sticks as levers to open enrichment devices |
| Cracking with stones/wood | All individuals use their teeth to crack open nuts and other objects. No observations of them using stones or wood to crack open objects. |
